# Computed Tomographic Evaluation of Buccal Shelf Dimensions in South Indian Patients With Sagittal Skeletal Class III Malocclusion: A Retrospective Study

**DOI:** 10.7759/cureus.43883

**Published:** 2023-08-21

**Authors:** Havisha Nookala, Swapna Sreenivasagan, Arvind Sivakumar, Aravind Kumar S

**Affiliations:** 1 Orthodontics and Dentofacial Orthopedics, Saveetha Dental College and Hospital, Saveetha University, Chennai, IND; 2 Orthodontics and Dentofacial Orthopedics, Reface Dental Hospital, Chennai, IND

**Keywords:** implant stability, growth pattern, extra-alveolar anchorage, class iii, mini screw, buccal shelf

## Abstract

Background

Computed tomographic evaluation of mandibular buccal shelf region in skeletal class III malocclusion cone beam computed tomography (CBCT) studies have been reported to have great alteration in the thickness of mandibular buccal shelf region owing to the different growth patterns and ethnic variations. The aim of this study was to determine the total and cortical bone thickness in the mandibular buccal shelf (MBS) region for extra-alveolar mini-screw placement in South Indian patients with sagittal skeletal class III malocclusion.

Material and methods

This retrospective computed tomographic study consisted of archived files of the Dravidian population with class III skeletal base that met the eligibility criteria. The total bone and cortical bone thickness of the buccal shelf regions were evaluated in relation to three anatomical sites at various depths and angulations. One-way ANOVA and Tukey honestly significant difference (HSD) post hoc tests were used for statistical analysis. Pearson correlation coefficient was performed to compare if any relation existed between bone thickness and the growth pattern.

Results

The maximum bone thickness in the buccal shelf region in our study was found at the distal portion of the second molar root, 8-12 mm from its cementoenamel junction (CEJ) and at 30-45 ° angulation (p-value<0.005). There was a positive correlation between the hypo-divergent growth pattern and the thickness of the bone.

Conclusion

Based on the sites recorded, the preferred site for mini screw placement in Class III patients is the distobuccal cusp region with respect to the second molar at a depth of 8-12 mm and at angulation of 30-45 °. There was a moderate correlation with hypo-divergent growth patterns, suggestive of a wider and thicker mandibular buccal shelf region in these subjects.

## Introduction

Sagittal skeletal class III malocclusion characterized by the sagittal discrepancy in the maxillomandibular relationship is attributable to either a prognathic mandible with an orthognathic maxilla or an orthognathic mandible with a retrognathic maxilla or a combination of both [1]. It is usually associated with a dentoalveolar compensation characterized by either proclination of maxillary incisors and retroclination of the mandibular incisors or both to maintain function [2]. This could even be associated with compromised facial aesthetics, which is one important reason for seeking treatment. In adult patients, non-surgical camouflage options are limited because of a lack of growth potential. However, adult patients with skeletal class III ranging from mild to moderate severity with acceptable facial aesthetics could really benefit from orthodontic camouflage treatment modality. It either includes an extraction pattern of bicuspids or en masse distalization of mandibular teeth, now possible with temporary anchorage devices (TADs) [3,4]. TADs which were traditionally used in cases that warranted asymmetric tooth movement in all three planes of space are now used as alternatives to orthognathic surgery to treat borderline surgical cases [5].

One of the common extra-alveolar sites for mini screw (MS) placement in the mandible to bring about en masse distalization is reported to be the mandibular buccal shelf (MBS) region [6]. According to the Misch classification for implant placement, the MBS region has a dense cortical bone (D2), with an implant success rate of up to 90%, thus proving to be a popular treatment modality [7]. It is reported that the most significant factors in predicting MS stability include bone thickness, mechanical retention, screw design, and placement technique [8]. Motoyoshi et al. reported that MS had a higher success rate when placed in an area with cortical bone thickness (CBT) ≥ 1 mm [9]. The prevalence of class III malocclusion in the South Indian population is about 2% [10]. However, multiple authors have reported anatomical variations in the MBS region in individuals based on their ethnicity and type of skeletal base pattern [11-13]. Despite numerous studies in different populations, there is still inconsistency in selecting the precise site for MA insertion. This could be attributed to the various local anatomical variations in the MBS region and the varying growth model of the individual, i.e., the vertical typology. Class III subjects are reported to have increased mandibular buccal bone thickness when compared to class I and II subjects [14]. A study by Ghosh et al. reported that MBS in the Indian population is mostly found to be thin and deep [15]. Therefore, the aim of this study was to determine the total (TBT) and cortical bone thickness (CBT) in the MBS region for extra-alveolar MS placement in the skeletal class III Dravidian population and to evaluate its correlation with the growth model of the individual.

## Materials and methods

This retrospective study involved cone beam computed tomography (CBCT) samples of South Indian patients with class III malocclusion, aged 18-30 years of age, who had reported to the Department of Orthodontics and Dentofacial Orthopedics from December 2018-January 2023 at Saveetha Dental College and Hospital for treatment. The study design was approved by the Institutional Human Ethical Committee (IHEC), Saveetha Institute of Technical and Medical Sciences (SIMATS) University and was granted IHEC number IHEC/SDC/ORTHO-2104/23/121

For sample size estimation, a priori test with a power of 0.90 and an alpha error of 0.05 was performed using G power analysis (G* power version 3.0.10, Kiel, Germany) to compute the required sample size by taking significant values of bone thickness across varying depths from our previous study by Sreenivasagan et al. [13]. The sample size was estimated to be 30 subjects. The archived diagnostic files of 60 skeletal Class III subjects who previously had reported to the department and sought treatment, were screened according to the following eligibility criteria; based on the digital cephalometric diagnosis performed using cephalometric software (Facad 3.6, Ilexis AB, Linköping, Sweden). Inclusion criteria included subjects with Class III malocclusion (ANB angle > 0° , Beta angle > 35°, Angle's class III molar relation/super class I molar relation); age group - 18 to 30 years; subjects who were indicated for CBCT before their orthodontic treatment. Patients with facial asymmetries and craniofacial anomalies; patients with systemic bone disorders; patients with missing teeth/prostheses in the posterior region or impacted teeth in the MBS region were excluded.

Methodology

**Figure 1:**
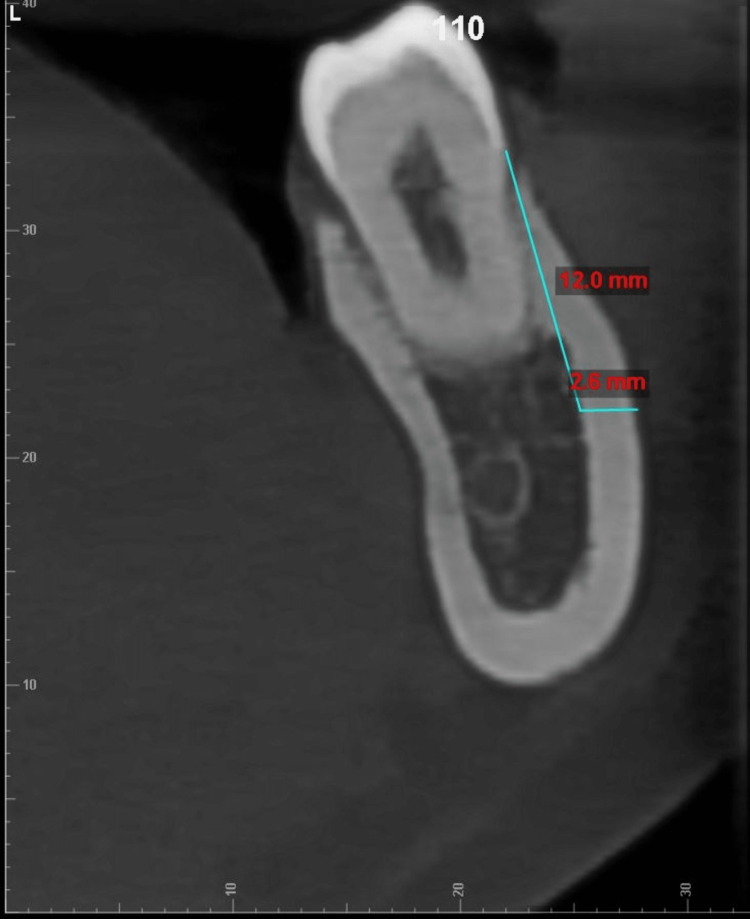
Example of measurement of total bone thickness at 12 mm depth from the CEJ of DB1M along its long axis CEJ - cementoenamel junction; DB1M - distobuccal cusp of permanent first molar

**Figure 2:**
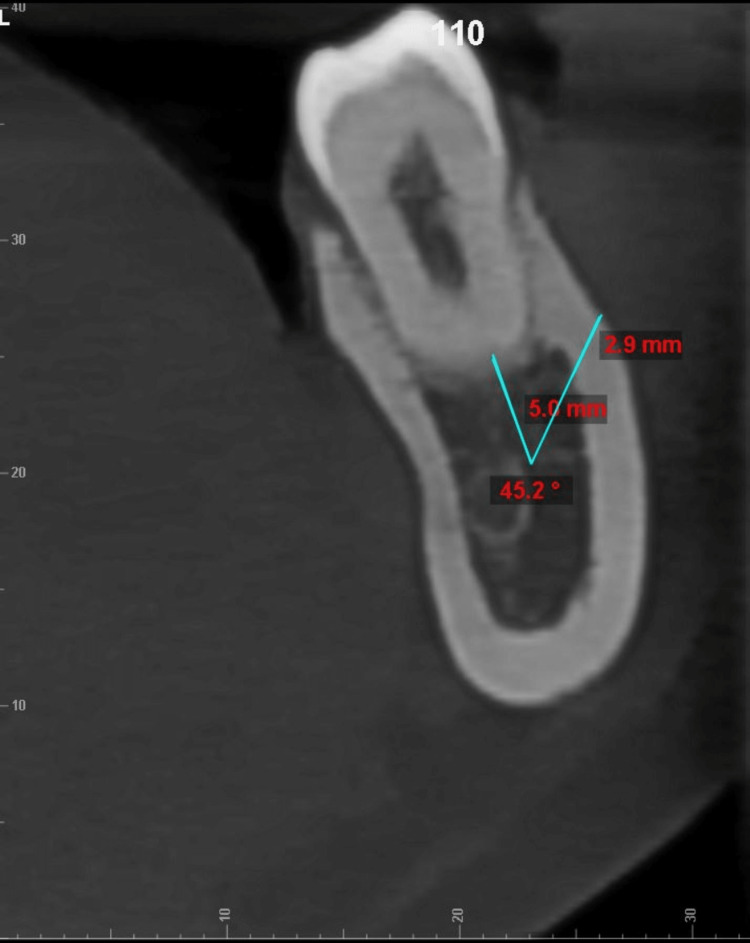
Measurement of cortical bone thickness measured at a point 5 mm from root apex at 45° angulation

**Figure 3:**
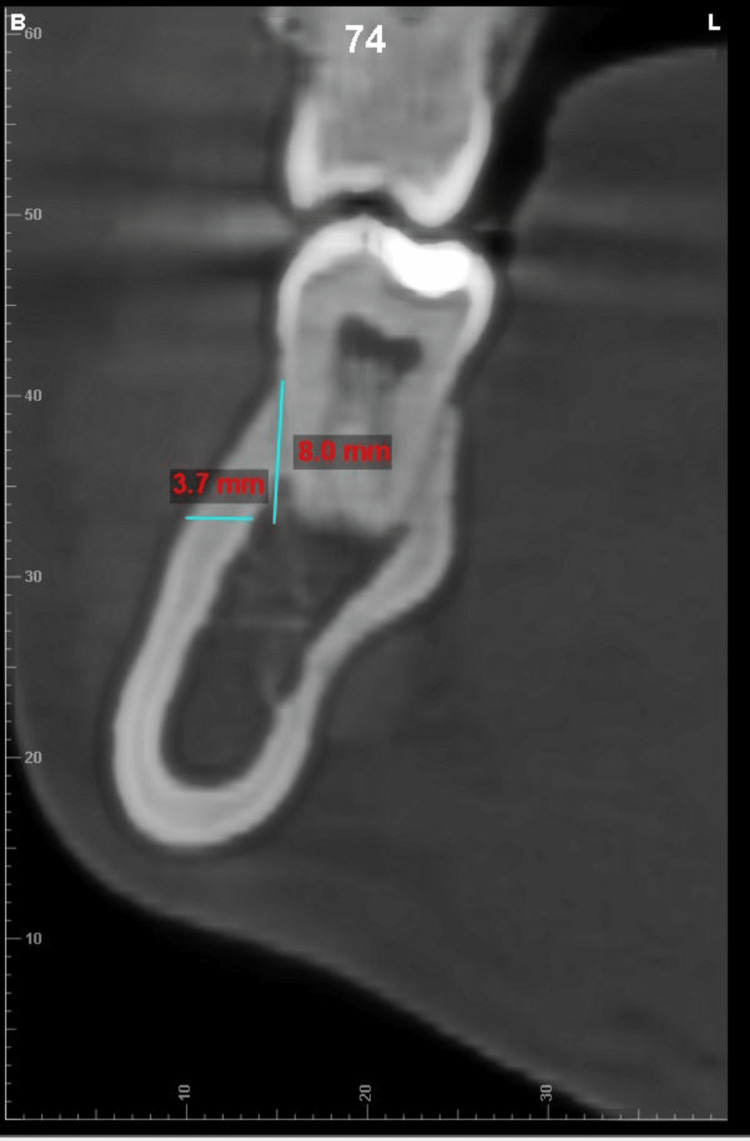
Example of measurement of total bone thickness at 8 mm depth from the CEJ of MB2M along its long axis CEJ - cementoenamel junction; MB2M - mesiobuccal cusp of permanent second molar

**Figure 4:**
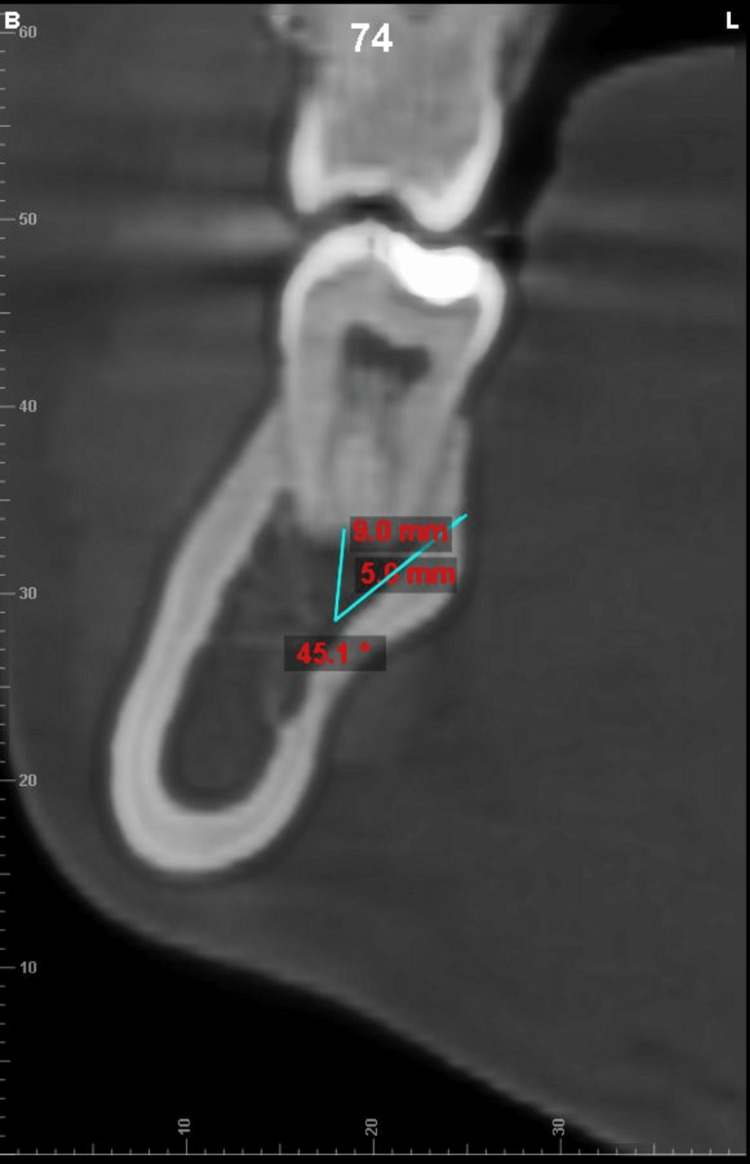
Measurement of total bone thickness (TBT) measured at a point 5mm from root apex at 45° angulation

**Figure 5:**
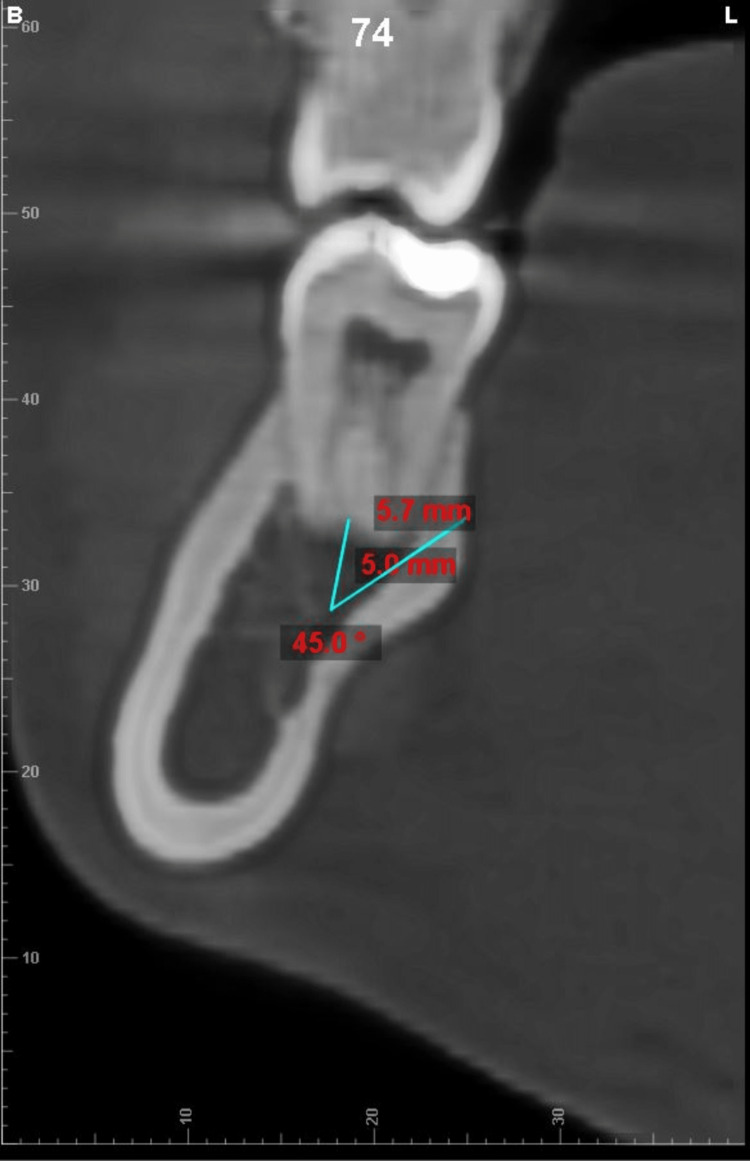
Measurement of cortical bone thickness measured at a point 5 mm from root apex at 45° angulation

All cone beam computed tomography (CBCT) records were obtained using the CareStream CS 9600 scanner (Carestream Dental, Rochester, New York) with the following parameters: 16 x 17 field of view cm; 120 kVp; 5mA, acquisition time: 24 seconds; and voxel size resolution: 150 μm. All CBCT scans were saved in Digital Imaging and Communications in Medicine (DICOM)-3 file format and imported and viewed in Dolphin™ software (Patterson Dental, Chatsworth, California). The CBCT was oriented along all three spatial planes. The axial plane was oriented along the furcation of the first and second mandibular molars, whereas the orientation of the sagittal plane was located along the central groove of the mandibular first and second molars. The coronal plane was oriented at the long axis of the tooth roots being examined. The lower cross-sectional view, set at a uniform slice thickness of 1 mm, was used for measuring the TBT and CBT. The measurements of TBT and CBT were made in both the hemiarches at the distobuccal cusp of the permanent first molar (DB1M) and second molar (DB2M) and the mesiobuccal cusp of the permanent second molar (MB2M) at depths of 4 mm, 8 mm and 12 from the cementoenamel junction along the long axis of the tooth (example illustrated in Figure 1). The TBT and the CBT were evaluated at different angulations of 30 °, 45 °, and 60 ° from a point placed 5 mm below the apex of the roots of DB1M, MB2M, and DB2M molars (example illustrated in Figure 2, 3, 4, 5). One examiner (HN) recorded all the required measurements. The slice images were randomly assigned numbers, and the examiner blindly re-evaluated the scans in a two weeks time interval to check for intra-operator concordance. Each measurement was taken three times, and its mean was considered for the final reading. Any conflict was resolved by discussion with two authors (SS, ASK). Steiner's mandibular plane angle, as assessed using Facad digital cephalometric software, was used to measure and categorically determine the growth pattern of each included patient (i.e., hypodivergent, hyperdivergent, and normodivergent), to study its correlation with TBT in the MBS region.

Statistical analysis

The statistical analysis was performed using SPSS statistics software version 23.0 for Windows (IBM Inc., Armonk, New York). The data was tested for normality using the Shapiro-Wilk test. The mean and standard deviation of the readings were determined by descriptive statistics. Statistical differences in TBT at various sites, depths, and angulations were determined by one-way ANOVA and Tukey honestly significant difference (HSD) post hoc test. Spearman's correlation coefficient was done to assess the correlation between the growth pattern and the TBT. The probability value (p-value) for statistical significance was set at 0.05. To determine intra-observer reliability, Kappa statistics was performed. Intra-operator concordances were evaluated on the basis of intraclass correlation, which was set ≥0.8. In case of any conflict of interest, it was resolved by discussion with the second author (SS).

## Results

The demographic data of the eligible subjects include patients from 18-30 years of age (mean age 23.5 years), categorized based on their growth pattern (n=10 hyperdivergent; b=20 hypodivergent). Kappa statistics showed an intraclass coefficient (ICC) value of 0.73 (95% CI: 0.69-0.74), which was suggestive of substantial agreement between the values. All data were normally distributed. The measurements of TBT and CBT at various depths and angulations were calculated according to the methodology mentioned above. According to these measurements, the bone thickness was measured maximum at the DB2M region. 

The descriptive statistics of the CBT at varying depths at the three anatomical sites are enlisted in Table 1. The DB2M region was recorded to have the maximum CBT at a depth of 12mm (2.939mm), followed by CBT at 8mm depth (2.636 mm). Minimum bone thickness was measured in relation to the DB1M region across all depths. The CBT at varying depths was statistically significant (p<0.01) at all three anatomical regions. The MB2M region at varying depths showed no statistical difference in CBT by the post hoc Tukey test. Significant differences in CBT and TBT were found at 30° and 45° (p-value<0.001) except at the MB1M region (Table 2).

**Table 1:** Descriptive statistics of cortical bone thickness at 4mm, 8mm, and 12mm distance from the CEJ of DB1M, MB2M, DB2M CEJ - cementoenamel junction; DB1M - distobuccal cusp of permanent first molar; MB2M - mesiobuccal cusp of the permanent second molar; DB2M - distobuccal cusp of permanent second molar

Cortical bone thickness		N	At 4 mm			At 8 mm			At 12 mm		
			Mean	SD	Sig	Mean	SD	Sig	Mean	SD	Sig
Distobuccal root of first molar (DB1M)	Right	36	1.975	0.758	0.805	2.531	0.6164	0.986	2.806	0.6004	1
	Left	36	2.181	0.788	0.981	2.417	0.681	0.8	2.747	0.4607	0.089
Mesiobuccal root of second molar (MB2M)	Right	36	1.983	0.7049	0.805	2.267	0.4997	0.986	2.531	0.5419	0.067
	Left	36	1.906	0.6551	0.889	2.358	0.5039	0.706	2.503	0.6331	0.002*
Distobuccal root of second molar (DB2M)	Right	36	2.217	0.7097	0.981	3.81	0.594	0.048*	4.4	0.4644	0.000*
	Left	36	2.067	0.7364	0.899	3.92	0.5592	0.046*	4.23	0.4987	0.000*

**Table 2:** Descriptive statistics of cortical bone thickness at DB1M, MB2M and DB2M 30, 45 and 60° at depth of 5 mm from root apex DB1M - distobuccal root of first molar; MB2M - mesiobuccal root of secnd molar; DB2M - distobuccal root of second molar

Cortical bone thickness		N	At 30		At 45		At 60		
			Mean	SD	Mean	SD	Mean	SD	Sig
Distobuccal root of first molar (DB1M)	Right	36	1.65	0.64283	2.2778	0.54341	2.2944	0.37816	0
	Left	36	1.3944	0.39775	2.4944	0.40873	1.9944	0.30769	0
Mesiobuccal root of secnd molar (MB2M)	Right	36	1.7611	0.49484	2.4889	0.51275	2.2267	0.59199	0.001
	Left	36	1.5778	0.40082	2.2389	0.49941	2.2333	0.53593	0
Distobuccal root of second molar (DB2M)	Right	36	1.6833	0.43148	3.73	0.37816	2.4056	0.39873	0
	Left	36	1.5556	0.59924	3.96	0.59199	2.4333	0.44211	0

The descriptive statistics of the TBT at varying depths and anatomic sites are enlisted in Table 3. The DB2M region was recorded to have the maximum TBT at 12mm depth (8.93mm) followed by TBT at 8mm depth (7.89mm). Minimum TBT was measured in relation to the MB1M region across all depths. The TBT across the varying depths was of statistical significance, as revealed by the post hoc Tukey test (p<0.01). The descriptive statistics of the TBT at varying angulations and sites are enlisted in Table 4. Maximum TBT was recorded at the DB2M region at 30° angulation (11.64mm), followed by TBT at 45° angulation (10.56mm). All sites except the MB1M region revealed significant differences in TBT at varying depths by the post hoc Tukey test (p<0.01). Minimum thickness at all the varying angulations was observed at the MB1M region.

**Table 3:** Descriptive statistics of total bone thickness at 4mm, 8mm, and 12mm distance from the CEJ of DB1M, MB2M, DB2M CEJ - cementoenamel junction; DB1M - distobuccal cusp of permanent first molar; MB2M - mesiobuccal cusp of the permanent second molar; DB2M - distobuccal cusp of permanent second molar

Total bone thickness	N	At 4 mm		At 8 mm		At 12 mm		Sig
		Mean	SD	Mean	SD	Mean	SD	
Distobuccal root of first molar (DB1M)	36	1.678	0.457	2.948	0.457	3.876	0.578	0
Mesiobuccal root of second molar (MB2M)	36	1.834	0.276	7.211	0.457	7.718	0.396	0.002
Distobuccal root of second molar (DB2M)	36	1.873	0.283	7.892	0.762	8.934	0.486	0

**Table 4:** Descriptive statistics of total bone thickness at 30 °, 45 ° and 60 ° distances from the CEJ of DB1M, MB2M, DB2M CEJ - cementoenamel junction; DB1M - distobuccal cusp of permanent first molar; MB2M - mesiobuccal cusp of the permanent second molar; DB2M - distobuccal cusp of permanent second molar

Total bone thickness	N	At 30 °		At 45 °		At 60 °		Sig
		Mean	SD	Mean	SD	Mean	SD	
Distobuccal root of first molar (DB1M)	36	9.164	0.241	9.492	0.274	9.749	0.277	0.051
Mesiobuccal root of second molar (MB2M)	36	9.471	0.138	9.469	0.294	10.638	0.237	0.003*
Distobuccal root of second molar (DB2M)	36	11.564	0.244	10.637	0.254	9.828	0.134	0.007*

The Pearson correlation test revealed a moderate positive correlation between the growth pattern and the bone thickness, suggesting that hypo-divergent subjects had a thicker bone thickness when compared to the hyper-divergent subjects (Table 5).

**Table 5:** Pearson's correlation test to determine any correlation between the bone thickness (based on depth) and the growth pattern

Parameter	N	Correlation coefficient	p-value
Hyperdivergent	10	0.077	0.08
Hypodivergent	20	1.000	0.011

## Discussion

The most common extra-alveolar site for the placement of mini screws is the MBS region, which is reported to show anatomic and ethnic variations. The objective of the study was to study the MBS dimensions among sagittal skeletal class III South Indian patients for the determination of the best possible site for MS placement and its correlation with facial growth patterns using CBCT scans. A retrospective study design was formulated, keeping in mind the as low as reasonably achievable (ALARA) principle for radiation safety and the Declaration of Helsinki. Only the computed tomograms of those patients were considered in the study who had been indicated for the above-mentioned radiogram during or before the course of their treatment and included were those who were diagnosed to have sagittal class III malocclusion. The sample size was estimated at 30 patients based on sample size estimation, with the power of the study determined at 0.95 and an alpha error of 0.05, from the TBT values from the study by Sreenivasagan et al. [13]. Multiple authors have advocated the use of CBCT scans in cases of skeletal discrepancy and justified its need for pre-treatment MS placement and in cases of complex anatomy so as to avoid impinging on neurovascular bundles [16-18]. The results of our study revealed a greater bone thickness at the distal portion of the second molar root when compared to its mesial portion. Both TBT and CBT increased with the increase in depth from the CEJ region of the first and second molar regions, with no significant difference in measurements in the right and left hemi-arches. The maximum CBT was in relation to the DB2M region at 12mm depth (4.42 mm) and at 45° (3.96 mm), and minimum at 4 mm depth in the DB1M region (1.975 mm).

Site of mini screw placement

The statistics revealed a greater bone thickness at the distal portion of second molar root when compared to its mesial portion, signifying that the distal region is a safer region for mini screw placement. The findings of our study are in accordance with studies by other authors who reported greater buccal bone thickness distal to the second molar [19-21]. However, Parinyachaiphun et al. reported greater CBT at the mesial aspect of the second molar than the first/second molar region [22]. 

Total and CBT at varying depths

Buccal CBT and TBT were measured at varying depths. The maximum bone thickness was reported in relation to the DB2M region. Bone thickness in right and left hemi-arches revealed no statistical difference. The maximum CBT was in relation to the DB2M region at 12mm depth (4.42 mm) and at 45° (3.96 mm) and minimum at 4 mm depth in the DB1M region (1.975 mm). A systematic review by Marquezan et al reports a positive association between CBT and mini screw stability [23]. A minimum CBT of 1.7mm and a minimum TBT of 5 mm was reported to be a prerequisite for a successful implant placement by Nucera et al [19]. Therefore, for successful miniscrew placement, the point of insertion must be at least 4 mm. Almost all sites had adequate CBT, ie. >2mm, suggestive of good stability for the placement of MS in these region. Mesial and distal regions of the 2nd molar bilaterally are the sites that showed more than 5 mm of total buccal bone thickness at 8 mm and 12 mm depth. Elshebiny et al studied the anatomy of the MBS by superimposing the STL models of the subjects on their CBCTs and virtually placing miniscrews after digitally tracing the inferior alveolar canal and also reported the greatest buccal and cortical bone depth distal to the 2nd molar at 8 mm from the CEJ [24]. Another study by Kolge et al studied the MBS at three different locations and two heights from CEJ and concluded that the DB2M region at 8 mm depth from the CEJ had the highest TBT (6.4mm) with the advocation of pre-drilling in cases of thick cortical bone which will result in the delayed failure of the miniscrew [25]. Trivedi et al. also reported greater CBT of 7 mm vertically from the alveolar crest at DB2M region and emphasizes caution while placing MS at this anatomic site due to its close proximity to the inferior alveolar nerve [20].

TBT and CBT at various angulations

The main concern while placing both intraarticular and extraarticular areas is to have optimum angulation for insertion to have maximum engagement with the bone and to avoid any contact with the adjacent molar roots. Of the measurements that were recorded at varying angulations of 30°, 45°, and 60°, the maximum TBT was measured at 45° (10.637 mm; p-value=0.000). The region of DBM1 showed the least amount of bone thickness. On the contrary, the study by Trivedi et al. reported maximum thickness at 30° angulation to the occlusal plane [20]. 

Correlation with growth patterns

The findings of the study indicate a moderate positive correlation between BT and the growth pattern, indicating that hypodivergent subjects had a thicker and wider bone thickness when compared to the hyper-divergent subjects. Vargas et al. also reported greater MBS bone thickness in short-faced individuals than in long-faced ones [21]. A study by Arvind et al. reported thicker CBT and bone density in hypo-divergent than in hyper-divergent subjects, similar to our findings [26]. A recent study reported a significant increase in posterior available space (PAS) at furcation in normodivergent subjects and suggested the mandibular posterior anatomic limit (MPAL) to mandibular arch distalization to be until the lingual cortex of the mandibular body [27]. In concordance with these findings, another study reported that mandibular MPAL significantly increased with a decrease in mandibular plane angle and with C-shaped root morphology, measured near the root apex at the cuspal line [28]. A recent study reported a larger retromolar space (RMS) for distalization in class III subjects and in cases with erupted third molars, resulting in a lower percentage of root contact due to the lingual inclination of the mandibular molars [29,30].

Limitations

A limitation of this study includes a smaller sample size, retrospective study design, and inclusion of a unicentric study population of specific South Indian ethnicity. MBS is reported to show anatomical variations with different ethnicities; therefore, larger-scale randomized clinical trials, including a larger sample size and with MBS insertion at varying depths and angulations involving multiple ethnicities, could be conducted for more precise results. Therefore, a heterogenous matched group of subjects in future studies would help us arrive at more precise and generalizable results. 

## Conclusions

The total and cortical bone thickness increased with the increase in depth from the CEJ region of the first and second molar regions in class III subjects. Based on the sites recorded, the preferred site for MS placement in the MBS region in Class III Dravidian patients was found to be the DB2M region, placed a the depth of 8-12 mm and at angulation of 30-45° to ensure its good engagement with the bone, thereby improving its stability. There was found to be a moderate correlation of MBS bone thickness with hypo-divergent growth patterns, suggestive of a wider and thicker mandibular MBS in these subjects.
